# Quick Identification of the Risk of Psychosis: The Italian Version of the Prodromal Questionnaire-Brief

**DOI:** 10.2174/1745017901814010154

**Published:** 2018-05-23

**Authors:** Antonio Preti, Andrea Raballo, Georgios D. Kotzalidis, Rosanna Scanu, Tamara Muratore, Mersia Gabbrielli, Debora Tronci, Carmelo Masala, Donatella Rita Petretto, Mauro G. Carta

**Affiliations:** 1 Center of Liaison Psychiatry and Psychosomatics, University Hospital, University of Cagliari, Cagliari, Italy; 2 Section on Clinical Psychology, Department of Education, Psychology, Philosophy, University of Cagliari, Cagliari, Italy; 3 Genneruxi Medical Center, Cagliari, Italy; 4 Department of Psychology, Psychopathology and Development Research, Norwegian University of Science and Technology (NTNU), Trondheim, Norway; 5 NESMOS Department, Sapienza University, School of Medicine and Psychology, Sant'Andrea Hospital, Rome, Italy

**Keywords:** PQ, Screening, Schizophrenia, Psychosis, Ultra high risk, Questionnaire, Distress score

## Abstract

**Background:**

Diagnosing people during the prodromal phase of an incipient psychosis can improve the chance of better outcome. In busy clinical settings, the ideal tool is a brief, easy-to-complete self-report questionnaire.

**Objective:**

To test the psychometric properties of the Italian version of one of the most used screening tools for the identification of the risk of psychosis, the Prodromal Questionnaire-Brief (PQ-B).

**Methods:**

Cross-sectional design. A convenience sample of college students was enrolled *via *snowball procedure (n=243; men: 45%). After understanding and signing the consent form, the participants received a booklet containing the following questionnaires: the 21-item Prodromal Questionnaire-Brief (PQ-B); the 12-item General Health Questionnaire (GHQ-12), and the 74-item Schizotypal Personality Questionnaire (SPQ). Receiver operating characteristic (ROC) analysis was used to assess the capacity of the PQ-B to identify individuals at risk of psychosis as independently defined based on the combination of GHQ-12 and SPQ thresholds.

**Results:**

The Italian version of the PQ-B revealed good internal consistency, test-retest reliability, and adequate convergent and divergent validity. The Youden method retrieved a cut-off = 7 for the PQ-B frequency score and a cut-off = 22 for the PQ-B distress score. Both PQ-B scores had a perfect (99%) negative predictive value.

**Conclusion:**

The PQ-B is a promising screening tool in two-stage protocols. The major advantage of the PQ-B is to exclude cases that are unlikely to be at risk of psychosis.

## 1 INTRODUCTION

The early intervention model in psychiatry posits that detection and treatment of people in their early stage of psychosis can greatly improve the course of the condition [1, 2]. There is some evidence that the time spanning from the initial manifestations of symptoms of psychosis, such as hallucination and delusions, and the start of an appropriate treatment with drugs and psychosocial interventions is related to the medium and long-term outcome of a psychosis in the spectrum of schizophrenia [3, 4]. The longer the duration of untreated psychosis, the poorer the outcome of the psychosis [3, 4].

Full-blown psychotic episodes are often preceded by a period of progressive impairment in social functioning, associated to nonspecific affective symptoms and attenuated manifestations of the symptoms that characterize the positive (hallucination- and delusion-like experiences) and negative (blunting anhedonia and apathy, autism-like social withdrawal) dimensions of the schizophrenia-spectrum psychoses [5, 6].

Diagnosing people during the prodromal phase of an incipient psychosis can improve its outcome. For this reason, several tools have been developed to allow the early detection of people with high at-risk mental states (HARMS) for psychosis, in order to increase their early access to treatment [7, 8]. A two-stage model is usually adopted with self-report screening followed by a clinical interview, this procedure has been proven accurate and efficient in other public health-care settings [9].

The Prodromal Questionnaire (PQ) is the most used tool for the initial screening of potential HARMS cases [10]. The initial version of the PQ included 92 items [11, 12]. Although, helpful for epidemiological purposes [13, 14], such a long version of the PQ may result cumbersome in the busy clinical setting. Therefore, two shorter versions have been developed: the Prodromal Questionnaire – brief version (PQ-B) [15], including 21 items, and a 16-item version of the Prodromal Questionnaire, the PQ-16 [16]. There is evidence that all versions of the PQ can reliably identify people at high risk of psychosis [10]. The PQ-B, in particular, showed good convergent and discriminant validity and test-retest reliability in US samples [17], excellent sensitivity to emerging psychosis, and strong agreement with clinician evaluations of attenuated psychosis symptoms in longitudinal studies [18, 19]. The PQ-B also provides measurement invariance across different ethnic groups, as demonstrated in multiethnic samples in the US [20]. Generally, higher cut-offs were required to identify cases at risk of psychosis in non-help-seeking samples than in general help-seeking populations, or in samples highly enriched with ultra high-risk participants [10].

The PQ and its shorter versions have been translated in various countries, including China [21], Spain [22], Nigeria [23], and Brazil [24]. The Italian version of the PQ-92 has been validated in a sample of 258 outpatients aged 11-36 years, who applied to the psychiatric mental health services in a large semi-rural area outside Rome [12]. No Italian version of the PQ has been evaluated in non help-seeking populations, which are the primary target of epidemiological surveys on the prevalence of psychosis-related symptoms and conditions.

This study illustrates the psychometric properties of the PQ-B in a sample of college students, a population at a higher risk of mental distress particularly during the first years due to the new pressures in the academic setting [25, 26].

## 2 METHODS

The institutional review board approved the study protocol in accordance with the guidelines of the 1995 Declaration of Helsinki, as revised in Tokyo in 2004, and further revised in Fortaleza, Brazil, in 2013 [27].

### 2.1 Participants

Young adults attending a university college in Center-South Italy were invited to take part in the study. The undergraduate sample was enrolled *via *a snowball procedure. Recruiters asked initial participants to take part in a study by completing a booklet and recruiting further participants among their colleagues, who were requested, in turn, to enroll other subjects, and so on. This method is designed to recruit a variegated array of individuals and avoid the bias of self-selection that occurs when recruiters choose from their personal social network only [28]. Anonymity was ensured.

We targeted a minimum sample size of 210 participants, 10 per each PQ-B item to assure adequate variance across the items. We increased the sample size to 300 to account for potential refusal. Out of 31,729 potential candidates among those attending university during the period of the study, 327 people were contacted: 26 declined after having a look at the booklet; 301 people accepted to fill in the questionnaire; 256 participants actually returned the booklet; 13 cases were rejected because their questionnaires were left blank in some essential parts (data on age or gender, or some items in two or more questionnaires); 243 participants were included in the study out of the 301 people who had accepted to participate (81%), and out of the 327 people who had been asked to take part in the study (74% overall participation rate). Because of the enrollment method (snowball procedure), we were unable to control from which faculty the university students involved in the study come from.

Participation was voluntary and no fee or other compensation was given for taking part in the study. All participants provided informed consent.

### 2.2 Measures

After having understood and signed the consent form, participants received a booklet containing the following questionnaires: the 21-item Prodromal Questionnaire-Brief (PQ-B); The 12-item General Health Questionnaire (GHQ-12), and the 74-item Schizotypal Personality Questionnaire (SPQ).

The PQ-B is a yes/no 21-item self-report questionnaire recording the positive symptoms experienced over the past month. For each endorsed symptom, responders rate whether they found it distressing or impairing, ranging from 1 (strongly disagree) to 5 (strongly agree), with 4 or 5 indicating distress. As a screening tool, the PQ-B is rated by using the total number of endorsed items (range: 0–21), the number of items that are identified as distressing (range: 0–21), and the total distress score (range: 0–105), which is the method recommended by Loewy and colleagues [15].

Standard procedures of translation and back-translation were used to translate the Italian version of the PQ-B from the original English version [29]. Translation accuracy was confirmed by an English-speaking translator and optimized with the help of the authors of the PQ and of the Italian translator of the PQ-92 (see Appendix for the Italian PQ-B).

The GHQ-12 is a screening tool aimed at identifying people in need of clinical attention [30]. The validated Italian version of the GHQ-12 was used in the study [31]. According to past studies, scores equal or above 4 on the GHQ-12 were considered indicative of clinically relevant psychological distress (*i.e.*, needing clinical attention [31]) However, patients with psychosis tend to score higher on the GHQ-12 than patients with anxiety or depressive disorders, and scores equal or above 6 better differentiate people diagnosed with psychosis from healthy people than the usual threshold of 4 [32]. Cronbach's *alpha* of 0.81 was found for the Italian validation study of the GHQ-12 [31]. The GHQ-12 has been translated and tested in a wide range of cultures and has proved itself a valid screening tool for common mental disorders in both Western and non-Western countries [33].

The SPQ is a 74-item self-report with a true/false format [34], which was developed to assess schizotypal personality disorder according to the Diagnostic and Statistical Manual of Mental Disorders-Revised, Third Edition (DSM-III-R) [35]. The Italian version of the SPQ was used in the study [36]. There is general agreement that the SPQ measures a multimensional construct, including a cognitive-perceptual deficits domain (ideas of reference, odd beliefs or magical thinking, unusual perceptual experiences, and suspiciousness subscales); an interpersonal deficits domain (excessive social anxiety, no close friends, constricted affect, and suspiciousness subscales); and a disorganized domain (odd or eccentric behavior and odd speech subscales) [37, 38]. The reproducibility of the first-order, nine-subscale structure of the SPQ and of its second-order domains has been demonstrated [39]. The SPQ has been translated into many languages, and there is wide evidence of its cross-national reliability and structural validity [40].

General socio-demographic information from self-report data was collected for the following variables: age, gender and socioeconomic status. To measure socioeconomic status we used the highest level of parental education [41], further subdivided into three categories, *i.e.*, lower than high school, high school diploma, college graduate or higher.

### 2.3 Statistics

In the database, there were no missing data, since any questionnaire lacking data in the essential part of the booklet was excluded (n=13). An independent research assistant rechecked the data after they were entered: Error rates were less than 1% and all were corrected following the questionnaires.

All data were coded and analyzed using the Statistical Package for Social Sciences (SPSS) version 20. Additional analyses were carried out in R [42].

All tests were two-tailed, with alpha set at p < 0.05.

Means with standard deviations were reported for continuous variables. Counts and percentages were reported for categorical variables. Parametric or non-parametric tests, as appropriate, were used to compare continuous variables between groups. Chi-square tests or Fisher's exact tests were used to analyze categorical data. Correlation coefficients were compared according to Steiger’s Z-test [43].

Scales reliability was measured by *Cronbach’s alpha*. For group comparisons, reliability values of 0.70 are considered quite satisfactory, and when dealing with subscales derived from a single questionnaire, values around 0.60 are considered acceptable [44].

Test-retest reliability of the PQ-B was evaluated in a subgroup of 120 participants, who were invited to complete the PB-Q again after 30 days. Participants included in the test-retest assessment were randomly recruited among those students whose university registration number was an odd number until reaching the quota of n=120.

Follow-up completion rate was 95% for the test-retest reliability sample (6 participants only did not return the booklet). Test-retest stability was assessed with the intraclass correlation coefficient (ICC), with 95% Confidence Interval (CI). The ICC is dimensionless statistics describing the reproducibility of repeated measurements in the same population: ICC values ≥ 0.60 are considered as acceptable for clinical use [45].

To assess agreement at retest for the PQ-B frequency score, we used the Bland and Altman [46] method (the PQ-B distress score depends on frequency scores, hence reproducing its Bland-Altman plot is unnecessary). The Bland-Altman plot visualizes the agreement between the scores of a test measured at two different assessment points by plotting the difference between test- and retest-scores against the mean of test- and retest-scores for each participant. Confidence intervals for the mean difference are calculated to determine if the latter deviates significantly from zero, which should not be. The plot draws the upper and lower limits of agreement, indicating the range within which 95% of the test scores in the two assessments can be expected to vary.

According to Raine [34], no more than a half of those scoring in the top 10 percent of SPQ would receive a diagnosis of schizotypal personality disorder. We assumed that those scoring in the top 10 percent of SPQ were more likely to have a schizotypal personality disorder and to be at risk of psychosis as well when they also manifested intense psychological distress. Subjects were identified as being High at-Risk Mental States (HARMS) when they scored above the cut-off ≥ 6 on the GHQ-12 [32] and scored in the top tenth percentile on the SPQ [34].

Receiver Operating Characteristic (ROC) analysis was used to assess the capacity of the PQ-B in identifying HARMS individuals as independently defined according to the combination of GHQ-12 and SPQ thresholds. Optimal cut-off points for frequency and distress scores were established according to the Youden method, using the *Optimal Cutpoints* package running in R [47]. ROC analysis was based on a logistic regression. The fit of the models was assessed with le Cessie - van Houwelingen - Copas - Hosmer unweighted sum of squares test [48], and Tukey-Pregibon test [49]. In these tests, the null hypothesis assumes that the model has a good fit, thus *p* < 0.05 (rejection of the null hypothesis) indicates misspecification of the model. The McFadden [pseudo]R^2^ and the adjusted McFadden [pseudo]R^2^ were used as a measure of explained variance, with values from 0.20 to 0.40 indicating good model fit [50]. Adjusted Odds Ratio (OR), with 95% confidence interval (95%CI), and estimated Wald test’s p were reported for each predictor in addition to the Area under the Curve (AUC). AUC threshold are: 0.80 to 0.90, good; 0.70 to 0.80, fair; <0.70, poor. ROC analysis was conducted with the *pROC* package running in R [51].

## 3 RESULTS

The sample included 109 participants who identified themselves as men and 134 participants who identified themselves as women (Table **1**). The age range was 19 to 34 years old, with mean age of 24.3 years (*SD,* 3.5; median, 24), with no difference by gender. No differences were observed in the distribution of PQ-B scores by age or socio-economic status, and a barely significant difference was found for the PQ-B distress score by gender, negligible in terms of effect size (Hedges’ *g*: -0.17; 95%CI: -0.42 to 0.09).

### 3.1 Internal Consistency and Test-Retest Reliability

Internal coherence, as measured by Cronbach’s alpha, was optimal for both the frequency and the distress scores of the PQ-B. Internal coherence was good to acceptable for the other scales and subscales that were used in the study (Table **2**).

Test-retest reliability for the PQ-B frequency scores, as measured by ICC, was 0.89 (95%CI = 0.86 to 0.92), and was 0.89 (0.87 to 0.91) for the PQ-B distress score.

**Fig. (1):**
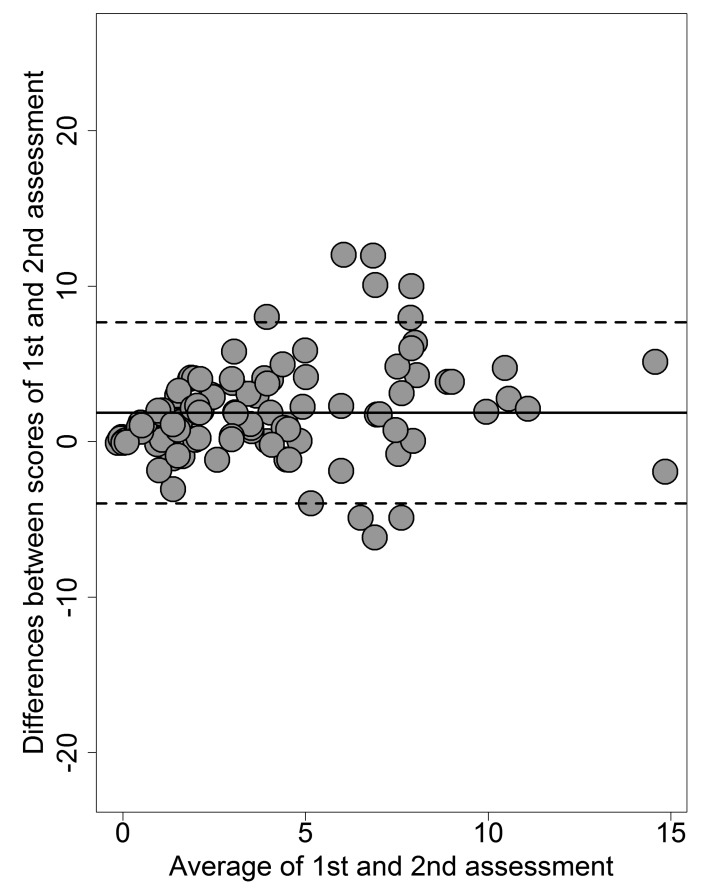
Bland-Altman plot of the PQ-B frequency score at test-retest assessment (n=114). The horizontal axis shows the mean of the two measurements ([test + retest]/2), while the vertical axis reports the absolute difference between the test and the retest (retest – test). Most values are expected to be within the 95% limit of agreement (average difference ± 1.96 standard deviation of the difference).

By plotting the differences and the means of the two assessments in the Bland-Altman plot, 7 cases only out of 114 were outside the upper and lower limits of agreement (Fig. **1**).

### 3.2 Distribution of Scores on the PQ-B

**Fig. (2):**
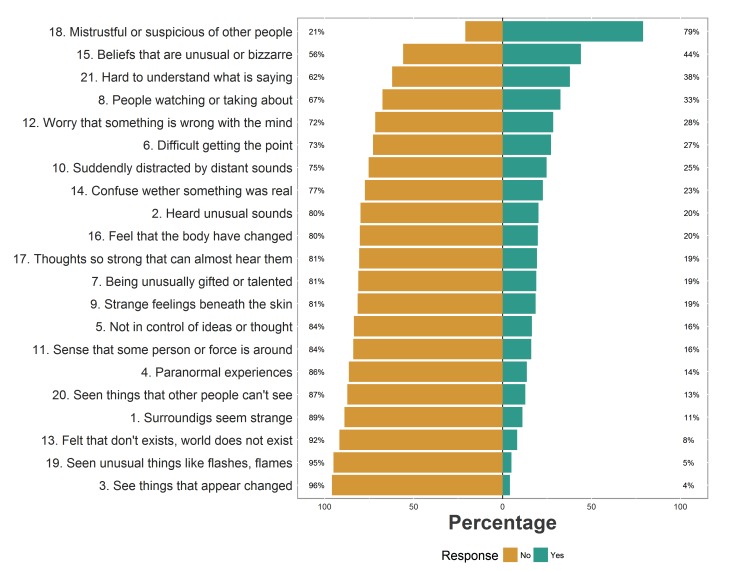
Distribution of scores by item for the Italian version of the Prodromal Questionnaire-Brief (PQ-B) in the sample (n=243). The items are numbered as they appear in the questionnaire, and are ranked according to the frequency of positive endorsement (response: yes).

Endorsement of items varied depending on the experience. Items concerning mistrustfulness and suspiciousness or unusual beliefs were endorsed by a large majority of participants, with only a minority endorsing items pertaining to visual hallucinations (Fig. **2**).

**Fig. (3):**
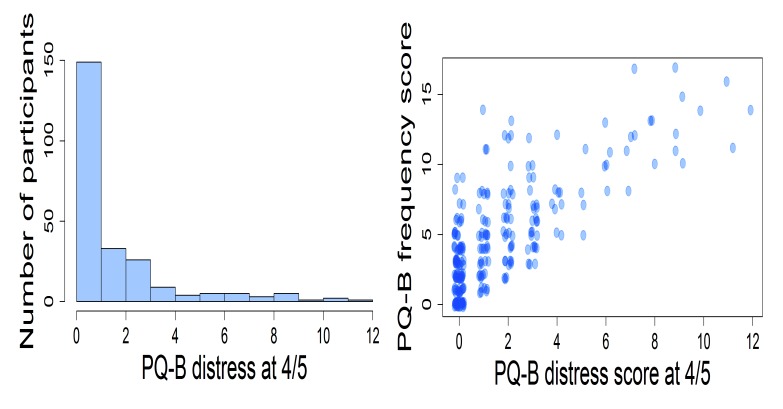
PQ-B distress score = 4 / 5 (positive psychotic experiences rated as distressful) and correlation with the PQ-B frequency score.

Participants endorsed an average of 5 positive psychotic experiences. The mean for the PQ-B distress score in the sample was 14. Only a minority of participants agreed that the experience was distressing (item rated 4 or 5). 42 participants (17.3%) rated just one experience as distressing; 33 participants (13.6%) rated two experiences as distressing, the remaining participants (n=61, 25.1%) rated three or more experiences as distressing. The experiences that were more often rated as distressing were those described in items 18 (mistrustfulness or suspiciousness of other people, 31%), 12 (worry that something is wrong with one’s own mind, 16%), and 21 (people sometimes finding it hard to understand what the subject is saying, 16%). The probability of rating a psychotic experience as distressing was related to the frequency of endorsement, but did not coincide with it; Spearman’s *rho* was 0.699, *p*<0.0001 (Fig. **3**).

### 3.3 Convergent and Divergent Validity of the PQ-B

Both the frequency and the distress scores of the PQ-B were related to psychological distress as measured by the GHQ-12. The PQ-B was positively correlated to SPQ subscales, but showed stronger links with the cognitive-perceptual deficits and disorganization domains than with the interpersonal deficits domains, or the measure of general psychological distress (Table **2**).

### 3.4 Predictive Ability of the PQ-B

In the sample, 59 participants (24.3%) scored ≥ 6 on the GHQ-12; 25 (10.3%) scored in the top tenth percentile on the SPQ, and 10 (4.1%) were identified as HARMS.

Both scores of the PQ-B were able to detect HARMS cases, with high accuracy (Table **3**).

The fit of the model in both analyses was optimal, with McFadden [pseudo]R^2^ > 0.20.

**Fig. (4):**
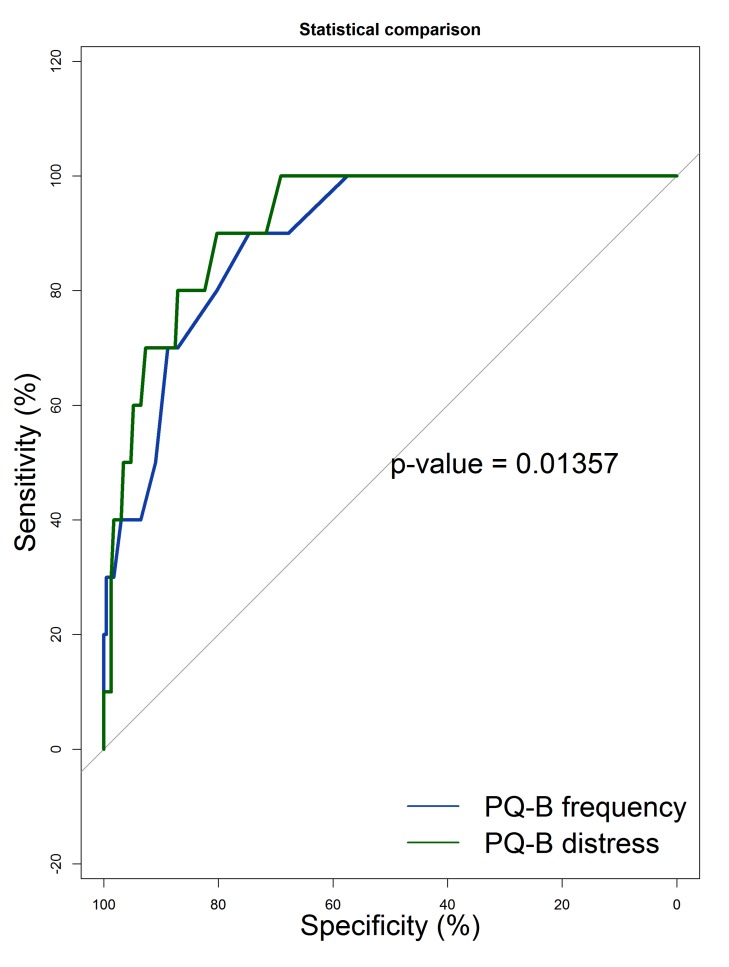
Comparison of the ROC curves between the PQ-B frequency score and the PQ-B distress score against the target, *i.e.*, high at-risk mental states (HARMS) cases as defined on the basis of the validated GHQ-12 and SPQ thresholds. The statistical significance of the difference between the areas under the two dependent ROC curves (derived from the same cases) with the method of DeLong *et al*. [58] is reported.

The PQ-B distress score had a better AUC than the PQ-B frequency score, with a small but statistically significant advantage (Fig. **4**).

The Youden method retrieved a cut-off = 7 for the PQ-B frequency score and a cut-off = 22 for the PQ-B distress score. Both the PQ-B frequency and distress scores had a very high negative predictive value (99%).

## 4 DISCUSSION

The Italian version of the PQ-B revealed good internal consistency, test-retest reliability, and adequate convergent and divergent validity. The predictive capacity of the tool is promising, and as in past studies, the PQ-B was able to detect people with HARMS with high accuracy, precision and performance (AUC close to 0.90).

It should be noted that the validity criterion that was used in this study is very conservative. The findings of this study should be intended preliminary as far as the predictive capacity of the tool is considered, since our criterion for HARMS case was entirely based on self-report tools. Findings of the present investigation need to be corroborated by further studies using a standardized interview as the gold standard. Nevertheless, the optimal threshold scores for the Italian PQ-B in this study were very close to the threshold suggested by Savill *et al,* [10] for general or mental health services in their comprehensive review of the studies where PQ was used as a screening instrument.

Noteworthy in this study is that positive psychotic experiences that were rated as distressful did correlate with their frequency of occurrence. Essentially, the more positive psychotic experiences the candidate reported, the more likely s/he had been distressed by these experiences. Thus, the frequency score of the PQ-B can be a reasonable summary score of the tool. Nevertheless, the distress score, as hypothesized by the authors of the instrument, is more accurate in detecting HARMS cases than the mere sum of the occurrence of the experiences (frequency score).

In this study, as in past studies, the major advantage of the PQ-B is to exclude cases that are unlikely to be at risk of psychosis rather than detecting potential cases at risk for further evaluation with a standardized interview. In a longitudinal study in the US, Kline *et al*. [18] concluded that an individual scoring below the recommended threshold score would be extremely unlikely to develop psychosis in the short or medium term. Albeit helpful, a “negative” screening tool requires a large-scale usage to produce a public health impact. To date, the PQ, in its various versions, has been used systematically to screen young adults seeking help from mental health services only in the Netherlands [52].

Despite these limitations, at the moment the PQ-B is the best screening tool for investigating the risk of psychosis in a two-stage scenario, for both epidemiological and clinical studies, and monitoring people with HARMS who are already in treatment [10, 53].

Due to its low positive predictive value, the better use of the PQ-B is probably in samples with a higher proportion of at-risk people (*e.g.*, help-seeking people, patients’ relative).

### 4.1 Strengths and Limitations of the Study

The assessment was conducted through self-report tools, which might have introduced some bias in responding, including the one related to social desirability. On the other hand, self-report measures allow the enrollment of large samples, and the guarantee of anonymity might have made participants more forthcoming when filling in the questionnaires. Unfortunately, we had not the opportunity to conduct a follow-up in order to further evaluate the people identified as being at potential (psychometric) risk with a dedicated interview, such as the Comprehensive Assessment for At Risk Mental States [CAARMS] [54], the Structured Interview for Prodromal Syndromes/Scale of Prodromal Symptoms [SIPS*/*SOPS] [55], or the Structured Interview for Prodromal Schizophrenia Proneness Instrument - Adult [SPI-A] [56]. The latter has shown better long-run ability to predict conversion to psychosis than the other structured interviews [57]. It should be noted that participants were undergraduates still attending university courses, and were therefore unlikely to have a full-blown episode of psychosis at the time of the study. However, since the participants were university/college students, the results cannot be immediately generalizable to the 19-34 year-old general population. We were unable to control which faculty/school did the university students involved in the study come from; thus, we cannot exclude that there has been some sort of sampling bias, for example, more students deriving from humanistic courses than from scientific ones. However, recruiters were specifically instructed to avoid sampling from psychology courses, to make sure that the knowledge of the topic did not introduce a bias in responding. Finally, we cannot exclude some sort of self-selection bias, inasmuch as those students who were more interested in the topic may have been also more prone to agree to participate in the study.

## CONCLUSION

This study confirms the promising psychometric properties of the PQ-B as a screening tool in two-stage protocols. The authors believe that its use could be helpful for both clinical and epidemiological purposes. For this reason, we have made available to the Italian clinicians and researchers the Italian version of the PQ-B and of the PQ-16 (Appendices A and B). Future studies could hopefully focus on comparing the two instruments to identify which is the most sensitive in excluding noncases.

## Figures and Tables

**Table 1:** General characteristics of the sample (n = 243).

Socio-demographic Group	N (%)	PQ-B Frequency Mean (SD)	PQ-B Distress Mean (SD)
Gender			
*Males* *Females*	109 (45%) 134 (55%)	4.7 (4.0) 4.9 (3.6)	13.0 (13.7) 15.2 (12.8) *
Age			
*19-24 years old* *25-38 years old*	137 (56%) 106 (44%)	4.9 (3.9) 4.6 (3.7)	14.9 (14.0) 13.2 (12.2)
Highest level of parental education			
*Compulsory school or lower* *High school diploma* *College graduate or higher*	104 (43%) 106 (44%) 33 (13%)	4.8 (3.8) 4.7 (3.8) 5.0 (3.8)	14.8 (13.3) 13.9 (13.1) 13.5 (13.8)

* Mann-Whitney U test: U = 6210.505; z = - 2.00, p = 0.045

**Table 2:** Mean scores on the measures of psychopathology used in the study, and inter-correlation among them and the PQ-B in the sample (n = 243).

	n. Items	Cronbach’s α	Mean (SD)	Median (IQR)	PQ-B *F*	PQ-B *D*
PQ-B						
*Frequency (F)*	21	0.83	4.8 (3.8)	4 (5)		
*Distress (D)*	21	0.85	14.2 (13.3)	10 (16)		
GHQ-12	12	0.85	3.4 (3.1)	3 (4)	**0.340**	**0.375**
SPQ Cognitive-perceptual deficits domain						
*Ideas of reference*	9	0 .77	2.2 (2.2)	2 (4)	0.598	0.592
*Odd beliefs or magical thinking*	7	0.64	0.9 (1.3)	0 (1)	0.497	0.531
*Unusual perceptual experiences*	9	0.63	1.3 (1.5)	1 (2)	0.571	0.574
*Suspiciousness*	8	0.75	2.4 (2.0)	2 (3)	0.521	0.551
SPQ Interpersonal deficits domain						
*Excessive social anxiety*	8	0.74	2.6 (2.1)	2 (3)	**0.391**	**0.410**
*No close friends*	9	0.61	1.0 (1.4)	1 (1)	**0.279**	**0.262**
*Constricted affect*	8	0.59	1.6 (1.5)	1 (3)	**0.345**	**0.329**
SPQ Disorganization domain						
*Odd or eccentric behaviors*	7	0.78	1.1 (1.6)	0 (2)	0.507	0.452
*Odd speech*	9	0.80	2.7 (2.4)	2 (3)	0.557	0.529

SD = standard deviation IQR = Interquartile range Pearson’s r p < 0.0001 in all correlations Estimates that differed from the others were reported in bold (Steiger’s p < 0.001)

**Table 3:** Receiver operating characteristic (ROC) analysis of the links between PQ-B and being a carrier of high at-risk mental states (HARMS) according to GHQ-12 and SPQ thresholds.

n = 243	PQ-B Frequency	PQ-B Distress
	OR (95%CI)	OR (95%CI)
PQ-B	1.47 (1.25 – 1.81); p<0.0001	1.11 (1.06 – 1.17); p<0.0001
True positive	9	9
False negative	1	1
Sensitivity	90.0%	90.0%
Specificity	74.7%	80.2%
Positive predictive value	13.2%	16.3%
Negative predictive value	99.4%	99.4%
Balanced accuracy	82.3%	85.1%
Diagnostic likelihood ratio	3.5	4.5
Fit of the model		
*Likelihood ratio test*	LR χ^2^=24.62; df=1; p<0.0001	LR χ^2^=26.59; df=1; p<0.0001
*AUC (95%CI)*	0.893 (0.815 – 0.971)	0.920 (0.857 – 0.983)
*McFadden’s pseudo-R^2^*	0.295	0.318
*McFadden’s adjusted pseudo-R^2^*	0.223	0.246
*Le Cessie-Van Houwelingen-Copas-Hosmer test*	z=-0.09, p=0.927	z=1.15, p=0.250
*Tukey-Pregibon test: Hat^2^*	z=1.47, p=0.142	z=-0.20, p=0.840
